# The Refined Composite Downsampling Permutation Entropy Is a Relevant Tool in the Muscle Fatigue Study Using sEMG Signals

**DOI:** 10.3390/e23121655

**Published:** 2021-12-09

**Authors:** Philippe Ravier, Antonio Dávalos, Meryem Jabloun, Olivier Buttelli

**Affiliations:** Laboratoire Pluridisciplinaire de Recherche en Ingénierie des Systèmes, Mécanique, Énergétique (PRISME), University of Orléans, 45100 Orléans, France; antonio.davalos-trevino@univ-orleans.fr (A.D.); meryem.jabloun@univ-orleans.fr (M.J.); olivier.buttelli@univ-orleans.fr (O.B.)

**Keywords:** multiscale, permutation, entropy, downsampling, electromyography, fatigue

## Abstract

Surface electromyography (sEMG) is a valuable technique that helps provide functional and structural information about the electric activity of muscles. As sEMG measures output of complex living systems characterized by multiscale and nonlinear behaviors, Multiscale Permutation Entropy (MPE) is a suitable tool for capturing useful information from the ordinal patterns of sEMG time series. In a previous work, a theoretical comparison in terms of bias and variance of two MPE variants—namely, the refined composite MPE (rcMPE) and the refined composite downsampling (rcDPE), was addressed. In the current paper, we assess the superiority of rcDPE over MPE and rcMPE, when applied to real sEMG signals. Moreover, we demonstrate the capacity of rcDPE in quantifying fatigue levels by using sEMG data recorded during a fatiguing exercise. The processing of four consecutive temporal segments, during *biceps brachii* exercise maintained at 70% of maximal voluntary contraction until exhaustion, shows that the 10th-scale of rcDPE was capable of better differentiation of the fatigue segments. This scale actually brings the raw sEMG data, initially sampled at 10 kHz, to the specific 0–500 Hz sEMG spectral band of interest, which finally reveals the inner complexity of the data. This study promotes good practices in the use of MPE complexity measures on real data.

## 1. Introduction

Electromyography is a relevant technique for providing functional and structural information about neuromuscular activities [1]. This technique also sheds light on motor learning [2] and neurological disorders [3]. Beyond this fundamental utility, surface electromyography (sEMG) application domains are diverse: there is the case of sports and ergonomics, whether performing isometric or dynamic exercises [4,5], as well as in clinical and technological applications.

The sEMG signal genesis consists of the activation of a large number of elementary functional entities called motor units (MU) [6]. A MU is composed of “the motoneuron and its adjunct muscle fibres” [7]. This MU activation called recruitment is one of the two modalities of the force control. The other one takes place by means of the rate coding [8,9].

MU activation is performed by a variety of motor units action potential (MUAP) [1] with different shapes whose mixture is the origin of sEMG. All of these constituting elements are governed by nonlinear interactions between each other, which makes the global dynamic behaviors difficult to predict from the elementary constituents. This confers to sEMG signals a notable level of complexity, including stochastic and deterministic components [10].

Numerous works using the sEMG technique have focused on changes in neuromuscular control in relation to the study of fatigue [11,12]. However, few works have explored complexity-based indicators [13]. The authors in [13] have presented a review of the complexity methods used in sEMG muscle fatigue studies. They noticed that some complexity-based indicators could efficiently detect manifestations of muscle fatigue. However, they highlighted the need for improvement of the reliability and sensibility of these indicators as well as the optimization of the parameter choice for the methods.

It is therefore interesting to evaluate more thoroughly and precisely the level of sEMG complexity by means of complexity-based measures. The entropy-based complexity measures have proven to be efficient tools for addressing complexity of such signals [14,15,16,17]. For example, in the recently published paper [17], the authors argued that the modified sample entropy measure appeared to be more sensitive to muscle fatigue and could yield more consistent results compared to the classical measures. The Permutation Entropy (PE) is also particularly a suitable tool for capturing useful information from the ordinal patterns of sEMG time series [15,18].

In [19], a study was achieved during dynamic contractions through an established protocol for fatiguing the deltoid muscle. This study demonstrated that the PE of sEMG signals had an interesting discriminant capacity between muscle fatigue, transition-to-fatigue and non-fatigue. This PE capacity slightly over-performed the classical sEMG median frequency feature. However, a limitation presented in [20] is that PE is confined to a single time scale; whereas, biological systems need to operate across multiple temporal scales. PE, therefore, does not capture all temporal interactions of the physiological control processes [21].

As an alternative to PE, the multiscale permutation entropy (MPE) [22] is a PE-based technique that allows exploring multiple time scales. This enhances detection of regularity and changes in muscle activity that can otherwise remain obscured by the fine resolution of the sampling frequency used for data acquisition. In [23], a fatigue study was addressed using MPE. It showed a significant difference between fatigue and non-fatigue cases: lower values of MPE were observed in fatigue cases.

Numerous PE variants that explore multiscaling were introduced in the literature, including refined composite downsampling PE (rcDPE) [24] and refined composite MPE (rcMPE) [25]. The superiority of the rcDPE on MPE and rcMPE was theoretically established in [24] by means of theoretical derivations of the estimator performance (bias and variance). We propose to go one step further and to study the performance of these techniques in a real context.

Therefore, the novelty of this article lies in the performance comparison of rcDPE, MPE and rcMPE estimators in the context of a fatigue study. All these considered techniques are applied to sEMG data acquired on the *biceps brachii* during a fatiguing static isometric contraction exercise. As the theoretical study presented in [24] demonstrated rcDPE prevailing over other techniques, we expect rcDPE to outperform both MPE and rcMPE in quantifying the fatigue level. Moreover, we particularly seek for the best scale value that allows for better discrimination of fatigue segments, and we discuss the link between this particular scale and the spectral analysis theory.

The paper is organized as follows. Section 2 presents a brief review of the background theory, including the definitions of PE, MPE and their refinements with a short insight in the statistical characterization. Section 3 details the experimental setup for data collection as well as teh statistical tests used for evaluating performance of the complexity estimators. In Section 4, the performance results are presented and discussed when applied to sEMG signals acquired under fatigue exercise. Section 5 sums up the study by proposing guidelines for the use of multiscale entropy estimators in the frame of sEMG fatigue studies.

## 2. Theoretical Framework

This section covers the necessary background for the PE-based methods. The original formulation of PE in [15] is presented as well as that of both MPE and Downsampling PE (DPE) and their refinements [22,24]. Subsequently, we provide a brief summary of the MPE statistical moments, which we originally developed in [26,27].

### 2.1. Permutation Entropy

Let us consider a real, weakly stationary and uniformly sampled signal $x={\left[x_{0},x_{1},\ldots ,x_{N-1}\right]}^{T}$ , where *N* is the sample number and *T* is the transpose symbol. For a given embedding dimension *d* and a given time delay τ , we represent the sub-sequence $x_{n},x_{n+\tau }\ldots ,x_{n+(d-1)\tau }$ by the coded sequence 1,2,3,…,d . The ordinal pattern of this sub-sequence is defined as the permutation of the coded sequence by sorting the sub-sequence values in an increasing order. For example, the possible ordinal patterns for d=2 and τ=1 are 12 ( $x_{n}<x_{n+1}$ ) and 21 ( $x_{n+1}<x_{n}$ ).

The probability distribution of patterns can be estimated by counting the sub-sequences of same pattern inside the signal x [15]. Therefore, let us design by $y_{i}$ the number of patterns of type *i* in the signal,

(1) $$y_{i}=\#\left\{n|\;n<N-\left(d-1\right)\tau ,\;\left(x_{n},x_{n+\tau }\ldots ,x_{n+(d-1)\tau }\right)\;\mathrm{is}\;\mathrm{of}\;\mathrm{pattern}\;\mathrm{type}\;i\right\}.$$

An estimate ${\hat{p}}_{i}$ of the true pattern probability $p_{i}$ can be obtained using $y_{i}$ :

(2) $${\hat{p}}_{i}=\frac{y_{i}}{N-(d-1)\tau },$$

Based on the probability mass function (pmf) formed by the pattern probability estimations of all possible patterns *i*, an estimation of the PE, denoted by $\hat{H}$ , can be obtained using Shannon’s entropy definition [15]:

(3) $$\hat{H}=-\sum_{i=1}^{d!}{\hat{p}}_{i}\ln{\hat{p}}_{i}.$$

Since the maximum entropy value is reached with the uniform distribution ( d! possible patterns with equal probabilities $p_{i}=\frac{1}{d!}$ ), a normalized version of (3) that guarantees an entropy value between zero and one is defined as follows:

(4) $$\hat{H}=\frac{\hat{H}}{\ln(d!)}=\frac{-1}{\ln(d!)}\sum_{i=1}^{d!}{\hat{p}}_{i}\ln{\hat{p}}_{i}.$$

PE is easy to implement and fast to compute, and it is invariant to nonlinear monotonous transformations [15]. This is an interesting property when the signals are subject to outliers. However, one should pay attention to the minimum signal length required to ensure sufficient pattern counts in (2) in order to provide an accurate estimation of the pattern distribution. Several recommendations were proposed for selecting the minimum signal length: N≫d! [15] or other less severe formulations, such as N≥5d! [28] or N>(d+1)! [29].

Since the PE technique only deals with a single set of data points, τ spaces apart, multiscale PE approaches were proposed to take into account the information content on long-range correlations.

### 2.2. Multiscale Permutation Entropy

We first present the MPE as it was introduced in [22]. We then recall MPE variants: composite MPE (cMPE) and refined composite MPE (rcMPE).

#### 2.2.1. MPE Formulation

The MPE procedure described in [22] can be considered as a two-step procedure [30]: (a) a moving average filter and (b) a downsampling. First, the signal x is partitioned in consecutive non-overlapping segments of size *m*. The average of each segment is then computed as

(5) $$x_{j}^{\left(m\right)}=\frac{1}{m}\sum_{i=m(j-1)+1}^{jm}x_{i},$$

where $\left(j,m\right)\in N^{2}$ . The obtained sequence $x^{\left(m\right)}={\left[x_{1}^{\left(m\right)},\ldots ,x_{N/m}^{\left(m\right)}\right]}^{T}$ is called a coarse-grained signal. Finally, the MPE is the PE of the coarse-grained signal $x^{\left(m\right)}$ calculated for different time scales *m*.

The coarse-graining procedure is useful for capturing trends or recurring patterns that are usually hidden in the ordinal comparisons between adjacent points of raw data. However, the main MPE drawback lies on its sensitivity to signal length as *N* decreases. The MPE bias and variance theoretically obtained in [24,27] show that the estimation of MPE is less reliable especially when the scales increase. The general constraint N/m≫d! must be fulfilled.

#### 2.2.2. Composite MPE

**Figure 1:**
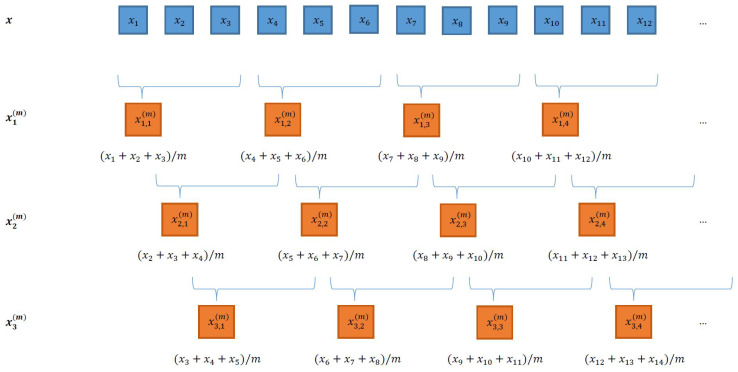
Principles of the composite coarse-graining procedure ( m=3 ). Nonoverlapping segments of size *m* are obtained from the raw signal. This leads to the original MPE estimation procedure using the single $x_{1}^{\left(m\right)}$ data vector. By shifting the initial positions, a set of coarse signals $x_{1}^{\left(m\right)}$ , $x_{2}^{\left(m\right)}$ , and $x_{3}^{\left(m\right)}$ is obtained. This leads to variant MPE estimation procedures using the set of *m* data vectors $x_{k}^{\left(m\right)}$ , with k=1,…,m .

By changing the starting element of the coarse-graining procedure, one can build a set of *m* different coarse grained signals $x_{k}^{\left(m\right)}$ as depicted in Figure 1. The *j*th sample of the $k^{th}$ coarse-grained signal for scale *m* is given by

(6) $$x_{k,j}^{\left(m\right)}=\frac{1}{m}\sum_{i=m(j-1)+k}^{jm+(k-1)}x_{i}.$$

For each composite coarse signal $x_{k}^{\left(m\right)}$ , the pmf of the d! patterns denoted by ${\hat{p}}_{k}^{\left(m\right)}$ is estimated, and the PE of $x_{k}^{\left(m\right)}$ denoted by $H_{k}\left({\hat{p}}_{k}^{\left(m\right)}\right)$ can be evaluated as

(7) $$H_{k}\left({\hat{p}}_{k}^{\left(m\right)}\right)=-\sum_{i=1}^{d!}{\hat{p}}_{k,i}^{\left(m\right)}\ln{\hat{p}}_{k,i}^{\left(m\right)},$$

where ${\hat{p}}_{k,i}^{\left(m\right)}$ is the *i*th component of ${\hat{p}}_{k}^{\left(m\right)}$ . Finally, for each *m* scale value, the cMPE is the average of the *m* obtained PE:

(8) $$H_{cM}=\frac{1}{m}\sum_{k=1}^{m}H_{k}\left({\hat{p}}_{k}^{\left(m\right)}\right).$$

Thanks to the shifting of the starting element and the larger number of coarse signals obtained, the composite approach counts new patterns that were not considered using the original MPE version. This allows the cMPE procedure to partially overcome the length constraints of the original MPE.

#### 2.2.3. Refined Composite MPE

For the purposes of building better empirical pattern probability distribution [31], the rcMPE uses a procedure that is different from the cMPE. Given a scale *m*, the rcMPE counts ordinal patterns for each composite signal $x_{k}^{\left(m\right)}$ , and it estimates their corresponding pattern probability ${\hat{p}}_{k}^{\left(m\right)}$ . A single and more robust pattern probability estimate ${\hat{\bar{p}}}^{\left(m\right)}$ can be defined using an averaging procedure:

(9) $${\hat{\bar{p}}}^{\left(m\right)}=\frac{1}{m}\sum_{k=1}^{m}{\hat{p}}_{k}^{\left(m\right)}.$$

At this point, the rcMPE is obtained by a unique entropy calculation using (9):

(10) $$H_{rcM}=-\sum_{i=1}^{d!}{\hat{\bar{p}}}_{i}^{\left(m\right)}\ln{\hat{\bar{p}}}_{i}^{\left(m\right)},$$

where ${\hat{\bar{p}}}_{i}^{\left(m\right)}$ is the *i*th component of ${\hat{\bar{p}}}^{\left(m\right)}$ .

By construction, the composite coarse-graining procedure produces signals with redundant information. This redundancy can impact the MPE statistics by increasing the bias and variance of the statistical properties of the raw signals [27]. In the following, we will refer to this effect as an artifact cross-correlation: by sharing common elements, the coarse signals are highly correlated, which can shadow the inherent dynamics of the raw signals.

In what follows, we present alternative PE variants that are exempt from this redundancy: downsampling PE (DPE), composite DPE (cDPE) and refined cDPE (rcDPE).

### 2.3. Downsampling Permutation Entropy

Recall that a time delay τ was introduced in the sub-sequence definition (1). The value τ=1 was considered until now. By taking τ≠1 , this time delay can be defined as a downsampling parameter [32] giving rise to the DPE measurement. In what follows, two DPE variants that improve the original DPE are reported.

#### 2.3.1. Composite DPE

**Figure 2:**
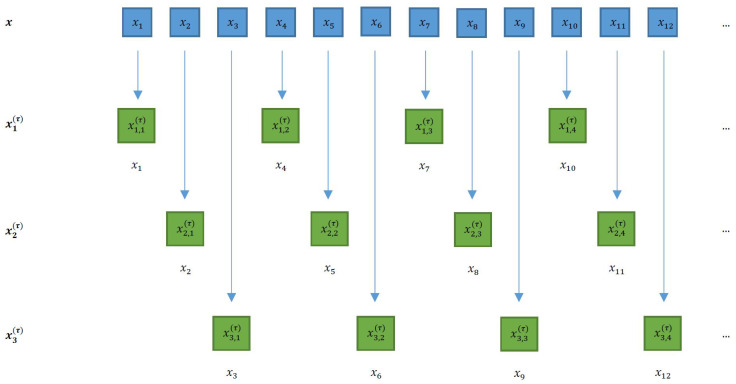
Principles of the composite downsampling procedure ( τ=3 ). Downsampled signals are obtained by taking data points spaced by τ . By shifting the initial positions between 1 and τ , a set of downsampling signals is obtained without any common data points.

By changing the starting element *k* of the downsampled procedure as shown in Figure 2, one can build a set of different downsampled signals $x_{k}^{\left(\tau \right)}$ with k=1,…,τ [33]:

(11) $$x_{k,j}^{\left(\tau \right)}=x_{k+\tau (j-1)}.$$

In the following, these obtained signals are called composite signals. For each composite signal, the pmf of the d! patterns can be obtained as ${\hat{p}}_{k}^{\left(\tau \right)}$ . For each value of τ , the cDPE is defined as the average of the PE of the obtained composite signals:

(12) $$H_{cD}=\frac{1}{\tau }\sum_{k=1}^{\tau }H_{k}\left({\hat{p}}_{k}^{\left(\tau \right)}\right),$$

with $H_{k}$ being estimated in the same way as in (7), changing *m* by τ .

#### 2.3.2. Refined Composite DPE

In [24], the combination of composite downsampling with a refined approach was proposed. Similarly to (10), the rcDPE is obtained by a unique entropy computation using a single pattern probability distribution ${\hat{\bar{p}}}^{\left(\tau \right)}$ ,

(13) $$H_{rcD}=-\sum_{i=1}^{d!}{\hat{\bar{p}}}_{i}^{\left(\tau \right)}\ln{\hat{\bar{p}}}_{i}^{\left(\tau \right)}.$$

${\hat{\bar{p}}}_{i}^{\left(\tau \right)}$ is the *i*th component of ${\hat{\bar{p}}}^{\left(\tau \right)}$ , which is the average estimation for each pattern probability over the composite downsampled signals. The pattern probabilities obtained by the cDPE and rcDPE procedures are different than the cMPE and rcMPE except for signals consisting of uncorrelated noise with uniform pattern distribution. This comes from the fact that we may have $p^{\left(\tau \right)}\neq p^{\left(m\right)}$ .

### 2.4. Statistical Performance of cMPE, rcMPE, cDPE and rcDPE

The authors in [34,35] did not mention artifact cross-correlation and did not discuss the statistical properties of the MPE, cMPE and rcMPE approaches. Thus, revisiting these concepts from this perspective was addressed in [24]. The explicit moments (bias and variance) of MPE, cMPE, DPE, cDPE and their respective refined versions was presented in [24].

Since redundancy between composite signals introduces an artifact cross-correlation effect, this makes exact derivations of the moments of the estimators of cMPE and rcMPE difficult. Conversely, we were able to enunciate the explicit moments of cDPE and rcDPE in [24], since the artifact cross-correlation effect is not present for the downsampled signals.

The explicit results in [24] demonstrated a decrease of the second term in the variance expression as a function of the scale values or the downsampling factor when applying the refined strategy. Moreover, the downsampling operation led to an approximated expression of the variance instead of an inequality expression for multiscale strategy.

Additionally, applying classical multiscale procedure to the composite signal construction is equivalent to applying a linear filter step before PE computation. This filtering step, which is not achieved in the case of downsampling procedure, may have a large impact on the estimators. For more details, readers can report to our previous work [36] where the linear filter’s intrinsic PE was characterized, and its contribution was separated from the signal’s ordinal information.

In the following, we will focus on MPE, rcMPE and rcDPE to investigate the superiority of the new rcDPE estimator on real datasets over the known MPE and rcMPE ones.

## 3. Methods

This section is dedicated to the performance comparison of MPE, rcMPE and rcDPE estimators on real datasets of sEMG signals acquired in a fatigue exercise. We first detail the experimental setup necessary to data collection. We then explain the data preparation to be used with the considered PE-based techniques. Finally, we present the statistical test we used to evaluate the performance of these techniques.

### 3.1. Experimental Setup

Data were collected during a previous study conducted by the signal team of the PRISME laboratory, in which ten healthy subjects (three women and seven men, ages 24 ± 1.5 year, all right-handed) participated in this study. They were fully informed about the experimental procedures, and every subject gave their signed consent.

An isometric ergometer was specifically designed for this experiment in order to perform an isometric flexion contraction of the elbow in a standardized position. Each subject was seated and belted at the waist and strapped at the shoulders. By means of a support, the right arm was horizontally rested (abducted perpendicularly from the body). The hand was in a neutral position (midway between supine and prone positions) with the elbow joint angle setting at 100° of extension and controlled with an electronic goniometer.

The force was measured by means of a strain gauge (0–2000 N force range, ZF, Scaime, France) and conditioned to reach a 1000-Hz frequency bandwidth (CMJCE analog conditioner, Scaime, France). The gauge was connected by a cable to a wrist-cuff attached proximal to the styloid process. The force level produced was displayed on a screen by means of a cursor. It was also possible to display a target for the force level to be imposed during the fatigue test.

The sEMG signals were recorded from the short head of the *biceps brachii* by means of electrodes located on the muscle belly halfway between the motor innervation point and the tendon after shaving and cleaning the skin. Raw sEMG signals were amplified by bipolar isolated amplifier (a common mode rejection ratio of 150 dB, 2–600 Hz band-pass filter). Force and sEMG signals acquisition were synchronized by an analog-to-digital card (PCI 6023E, National Instrument, Austin, TX, USA) at a 10 kHz sampling frequency.

The protocol consisted of three parts involving isometric elbow flexion contractions. Step 1 was a standard warm-up of low intensity and very short duration contractions. In step 2, after two minutes of rest, three maximal contractions of less than 3 s were performed in order to determine the maximal voluntary contraction (MVC), which allowed the contraction intensity level of the fatigue test to be set, and then 3 min rest was imposed between contractions. In step 3, the fatigue test was performed by the subject. They were asked to maintain contractions until exhaustion at 70% MVC. By exhaustion, we refer to the point at which the subject was no longer able to maintain a constant level of force.

### 3.2. Data Setup and Entropy Techniques

We explore the considered PE-based techniques as a function of the *fatigue* factor by carrying out calculations on the data sets obtained with sustained force until exhaustion. Each recorded signal obtained during this work is split into four non-overlapping segments of equal length, labeled as windows ( $W_{1}$ , $W_{2}$ , $W_{3}$ , $W_{4}$ ), in chronological order (0–25%, 25–50%, 50–75% and 75–100% time to exhaustion, respectively).

MPE, rcMPE and rcDPE techniques were applied to each segment. We tested different values of scale (*m*) from 1 to 100 in one-step increments. We also checked different dimension values (*d*): 3, 4 and 5. Since the recorded signals were around 274,000 data samples, the maximum dimension of analysis was set at d=5 , and the length criterion adheres to the aforementioned discussion on classic MPE (Section 2.2.1). For practical purposes, the downsampling parameter for rcDPE was set at τ=m .

### 3.3. Statistical Tests

The average of each considered entropy estimator was calculated on ten values for each of the conditions presented in this study: scale, dimension and fatigue step. The difference between methods, their parameters (scale and dimension) as well as the difference between the segments were evaluated by using gross results from segment $W_{1}$ to $W_{4}$ under the fatigue condition. For the *fatigue* (delta step) condition, five combinations were taken into account: [ $W_{1}-W_{2}$ ; $W_{1}-W_{3}$ ; $W_{1}-W_{4}$ ; $W_{2}-W_{3}$ ; $W_{3}-W_{4}$ ].

The statistical analysis was performed by means of the Statistica 7.1 Software (Stat Soft. Inc., Tulsa, OK, USA). Given that samples are drawn by normality and equal variances in all groups (evaluated by the Lilliefors and the Levene tests [37], respectively), statistics were conducted by means of three-way repeated measures analysis of variance (ANOVA).

The repeated measure was the step fatigue (or the delta step fatigue) and corresponded to the first factor. The other two factors were the method (MPE, rcMPE and rcDPE) and the dimension (3, 4 and 5). When a significant difference was observed, the Bonferroni comparison procedure was used in order to isolate the differing groups. All significance thresholds were fixed at α<0.05 .

## 4. Results & Discussion

We first present and compare the results of the considered methods and their settings when applied to sEMG signals acquired under fatigue exercises. We then discuss these results and compare them to the existing literature of fatigue studies.

### 4.1. Comparison between Methods and Their Settings

**Figure 3:**
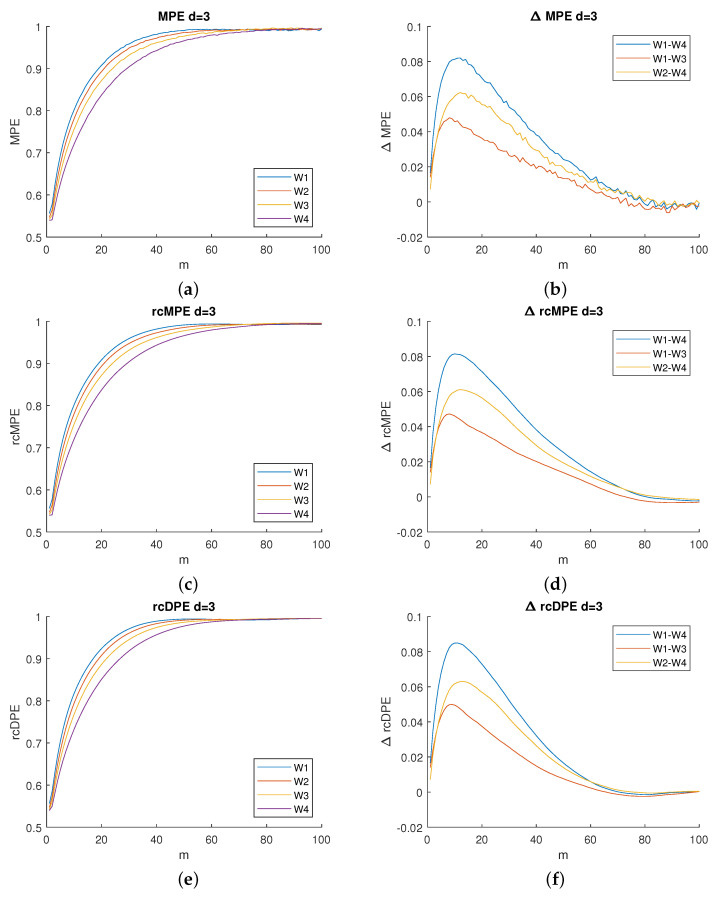
Left column: mean entropy values as a function of scale *m* for the fatigue steps (from $W_{1}$ to $W_{4}$ ). Right column: variation in entropy mean values as a function of scale *m* on the pairwise differences between steps of fatigue (only $W_{1}-W_{3}$ , $W_{2}-W_{4}$ , and $W_{1}-W_{4}$ are shown). From top to bottom: MPE (**a**,**b**), rcMPE (**c**,**d**) and rcDPE (**e**,**f**). All values at dimension *d* = 3.

Figure 3 displays the results obtained using the three methods: MPE, rcMPE and rcDPE. In the left column of this figure, four curves are plotted, and each curve corresponds to the mean MPE plotted as a function of time scale *m* at dimension d=3 for four window segments ( $W_{1}$ to $W_{4}$ ). Two behaviors can be observed in these curve kinematics.

A rapid increase is first noticed in the shorter time scales (from m=1 to m≈60 ), followed by a steady state ( m>60 ) for the three considered PE-based techniques. The four curves belonging to the four segments differ below this value m=60 and are superimposed above it. Regarding the methods, the curve profiles are similar, although both of the two refined composite methods show smoother lines in comparison to MPE.

The right column of Figure 3 displays the difference of the mean entropy values calculated using pairwise of the fatigue steps $W_{1}-W_{3}$ , $W_{2}-W_{4}$ and $W_{1}-W_{4}$ . This difference ( ΔMPE ) is plotted as a function of the scale *m*. This representation allows us to refine the scale selection that serves as the best differentiation between segments. Indeed, the curves present a significant difference for values of *m* ranging from 8 to 15.

**Figure 4:**
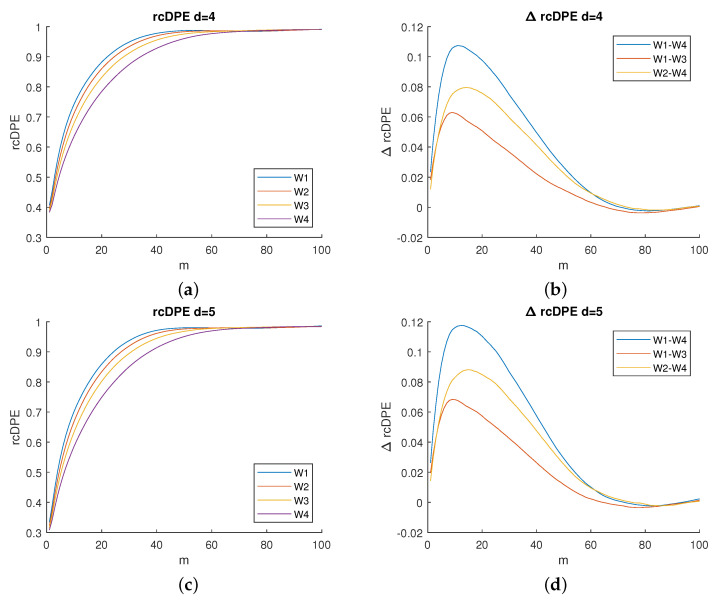
Left column: mean entropy values as a function of scale *m* for the fatigue steps (from $W_{1}$ to $W_{4}$ ). Right column: variation in entropy mean values as a function of scale *m* on the pairwise differences between steps of fatigue ( $W_{1}-W_{3}$ , $W_{2}-W_{4}$ , and $W_{1}-W_{4}$ ). Dimension values, from top to bottom: d=4 (**a**,**b**) and d=5 (**c**,**d**).

As this range remains unchanged regardless of the method applied, we can deduce that it corresponds to the optimal scale range of analysis for this particular dataset. This range remains unchanged even if a higher embedding dimension is selected as illustrated in Figure 4. Therefore, we keep the m=10 for the subsequent steps in our analysis.

Additionally, when comparing the entropy variation values depicted in Figure 3f and Figure 4b,d, we notice that the maximum dimension used ( d=5 ) yielded the highest differences between segments. We did not use dimensions higher than five in order to maintain the MPE bias below acceptable levels due to the signal length constraints [27]. This restriction does not apply to rcMPE and rcDPE, where we can use higher dimensions [27]. However, we should pay attention to the increasing computational costs if higher dimensions are used.

**Figure 5:**
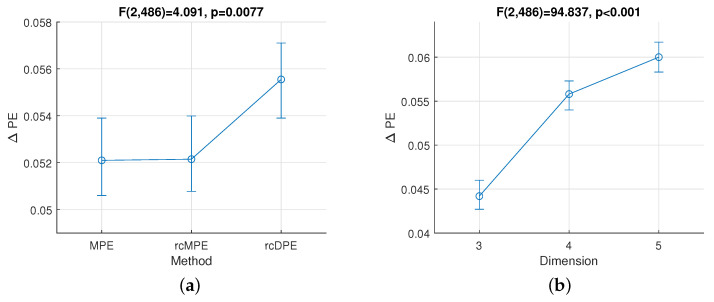
Effective hypothesis decomposition for fatigue isometric contraction for the following factors: (**a**) PE-based methods and (**b**) dimension. The vertical lines denote 95% confidence intervals for the ANOVA *F* distribution.

According to the ANOVA statistics, the rcDPE method better distinguishes between the fatigue activity segments at isometric contraction compared with the two other considered methods as illustrated in Figure 5a. Indeed, the method factor, shown in Figure 5a, has a significant effect on the ΔMPE value ( F(2,486)=4.091 , p=0.0077 ). The rcDPE produces notable differences by comparison with the other two methods MPE ( p=0.017 ) and rcMPE ( p=0.025 ).

The importance of the embedding dimension in the differentiation between entropy values is highlighted in Figure 5b as well. As shown in Figure 5b, the dimension factor has a significant effect on the ΔMPE value ( F(2,486)=94.837 , p<0.001 ); the dimension d=5 produces higher differences than d=4 ( p<0.001 ) or d=3 ( p<0.001 ). However, no evidence of interaction between factors has been reported.

### 4.2. Physiological Fatigue Study

**Figure 6:**
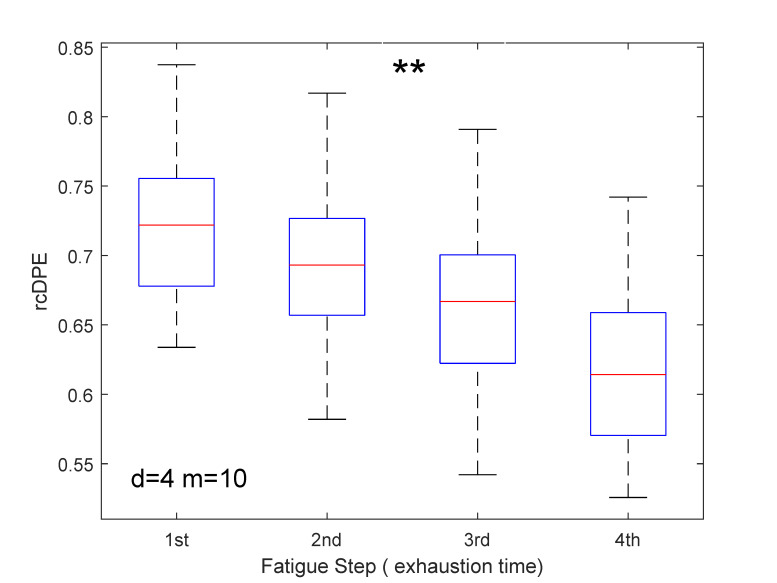
Range boxplots for rcDPE measurements from isometric contractions with dimension d=4 . Four activity windows from sustained 70% MVC at scale m=τ=10 . From the repeated ANOVA test measurements, all the pairwise comparisons are statistically significant ( p<0.001 ), shown globally with **.

The physiological application was managed using the rcDPE technique by selecting the dimension d=4 and scale m=10 for the sustained fatigue exercise condition. We remind that ten subjects have participated to this study. Figure 6 displays the rcDPE variation plotted against the segment rank of fatigue level. It can be noticed from Figure 6 that there is a significant rcDPE decrease with time progression to exhaustion that occurred throughout all stages ( p<0.001 ). Additionally, repeated ANOVA measurements concerning the fatigue factor revealed statistically significant effects of the rcDPE ( F(3,27)=72.3 , p<0.001 ).

### 4.3. Discussion

We first discuss the impact of PE parameters when applying PE-based techniques to sEMG signals. We are therefore able to propose optimal parameters values based on the observed results under fatigue conditions. Second, we explain the benefits of the rcDPE method. Third, we discuss the optimal parameters at the light of spectral analysis theory when applied on sEMG data. We finally make connections to the existing literature of fatigue studies.

It must be noted that, in the current discussion, we neglect the bias of the considered PE estimators even at high time scales since the signal length constraint can be assumed to be satisfied [24,27]. Indeed, the selected dimension is below five, and the length of the collected sEMG data is above N= 274,000 samples. This high signal length yields a small MPE variance, which contributes little to the overall observed variability between subjects [24].

#### 4.3.1. Optimal Parameter Settings

Based on the observed results (Figure 3f and Figure 4b,d), we notice that choosing a time scale that is either low or too high would affect the experiment. Indeed, choosing a time scale that is too small would make the differences between methods almost indistinguishable, whereas a time scale that is too high would make all of the methods useless since they cannot distinguish sEMG complexity from noise.

Moreover, for the sEMG signals recorded under fatigue exercise, we found the time scale with the most pronounced difference between segments was around m=10 (or τ=10 ). Since the original sampling frequency used for the experiment was $f_{s}=10$ kHz, the effective sampling rate after coarse-graining or downsampling is $f_{s}=1$ kHz, which only captures information in the range 0–500 Hz as a consequence of Shannon’s theorem. We thus expect that the most adequate sampling rate for PE calculations will be close to 1000 Hz.

#### 4.3.2. Benefits of the rcDPE Estimator

The benefits of rcDPE can be explained by the combination of the composite procedure with downsampling. Indeed, the composite procedure increases the number of time series from the raw data. Then, the averaging steps (8) or (9) help to reduce the noise impact on the estimators and highlight coherent structures. Thus, applying the composite strategy may lead to a reduced sensitivity to noise of the estimators.

In the case of the coarse-graining procedure, the averaging benefit is limited due to the presence of cross-correlation artifacts. These artifacts are artificially induced by redundant elements due to the coarse-graining procedure itself. This explains the results of Figure 5a showing no difference between MPE and rcMPE in terms of fatigue level discrimination, even if a smoothing effect is present in Figure 3d compared with Figure 3b.

In the case of the downsampling procedure, this artifact cross-correlation effect is not present. Therefore, as long as the Shannon theorem is fulfilled (no information is loss), downsampling combined with the composite procedure increases the number of time series from the raw data, by generating components free of cross-correlation artifacts. This helps to decrease the variance of cDPE and rcDPE estimators, which is in agreement with the theoretical statistical properties previously established in [24,27]. This also explains the results of Figure 5a showing differences between (MPE, rcMPE) and rcDPE in terms of fatigue level discrimination.

#### 4.3.3. Link with Spectral Analysis Theory

Regarding the spectral theory, PE measures can be categorized into two classes with respect to the scaling effects.

As a first class, the MPE measure is achieved by a coarse-graining procedure on nonoverlapping segments of size *m*. The effect of this scaling operation is equivalent to a classical filtering of the data using an *m*-constant coefficients filter followed by an *m*-downsampling operation. For example, by choosing m=10 , multiscaling acts as a 10-tap constant value finite impulse response filter with a cutoff frequency of $f_{c}\approx$ 445 Hz, and the sampling frequency is $f_{s}=10$ kHz. Even if this natural lowpass filter can prevent spectral aliasing, it is still a poor quality filter [38], and the information loss can impact the rcMPE performance. We deeply discussed the effect of this linear intrinsic filter preprocessing in our previous paper [36].

As a second class, the DPE measure is achieved by a single downsampling operation. We kept this original definition in the paper while being aware of the spectral aliasing. Downsampling without any prefiltering produces spectral aliasing, and many authors do not mention this. In our study, the aliasing effect can be neglected up to time scale 10 through two points.

Firstly, in our experiments, we carefully acquired data with very limited high-frequency noise using high-quality sensors and high-performance instrumentation under controlled laboratory conditions. Secondly, the physiological sEMG activity lies below 300 Hz regardless of the muscles investigated. This is the frequency range where sEMG information is produced by the muscle fiber contractions [39].

**Figure 7:**
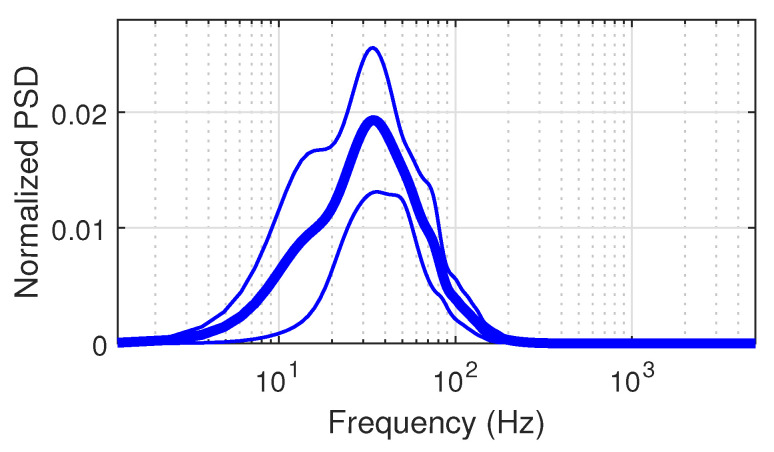
PSD averaged over the ten studied subjects (thick line) with the standard deviation (thin lines) in *x*-axis semilog representation. Integrated normalized PSDs are in unity.

**Figure 8:**
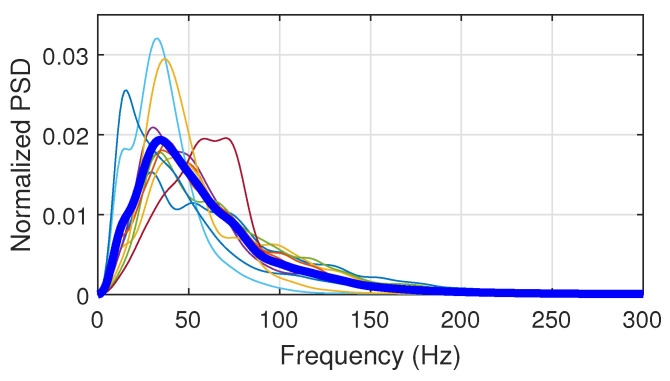
PSD for the ten subjects in a linear representation. The thick line is the averaged curve over the ten subjects. Integrated normalized PSDs are in unity.

Figure 7 displays the semilog plot of the average and standard deviation of power spectral densities (PSD) calculated using the acquired sEMG signals of ten studied subjects. The linear representation of the PSD is also depicted in Figure 8. Both figures illustrate the low level of noise in the high frequency band.

In order to numerically support this claim, we evaluated the power ratio defined as the ratio between the integrated PSD in the *sEMG band* [0;500 Hz] and the *noise band* [500;5000 Hz]. This ratio is 29.5 dB, which highlights a negligible noise level compared to power of the sEMG spectral band of interest. Therefore, the spectral aliasing due to the downsampling with τ≤10 can be neglected.

Based on this, and since the downsampling with τ=10 reduces the sampling frequency to 1 kHz, we can assume that the sEMG activity information will be concentrated in the frequency bandwidth of interest without noticeable distortion. At τ>10 , the spectral aliasing begins to be noticeable, which destroys the real frequency structures, thus making rcDPE inappropriate at such scales. Both estimators tend to white information measure especially when m>20 .

The link to the spectral analysis theory is, therefore, of high importance for an informed and correct interpretation. As a general rule of thumb when working with sEMG signals in the context of a *biceps brachii* fatigue study at a static isometric 70% MVC contraction level, we recommend the use of a time scale that adjusts the coarse (or downsampled) signals close to 1000 Hz with at least an embedding dimension close to d=4 .

Generally, these PE parameter values (*d* and τ for rcDPE or *m* and for rcMPE and cMPE) will not be standard and will depend on the particular characteristics of the dataset and the application domain. They should be adjusted until a maximum difference between groups is reached, provided that Shannon’s theorem is respected. Notably, the recommendation is to limit the observed data inside their full frequency band of interest in order to achieve higher sensitivity with the rcMPE or rcDPE methods.

#### 4.3.4. Physiological Findings Compared to Existing Fatigue Studies

Regarding our contribution to physiological fatigue studies, the entropy decrease observed (Figure 6) may be explained by physiological modifications that are performed during the sustained isometric exercise. Indeed, during such an exercise, peripheral disturbance in muscle contraction has been noted and linked to fatigue phenomena [40]. In particular, an increase in the duration of action potential was observed [41] as well as a consequent increase in the time duration of the MUAP [42]. The lengthening of the time duration may make the probability distribution of ascending or descending patterns increase, thus, unbalancing the distribution, which leads to lower entropy values.

Moreover, MU firing rate synchronization may also explain the entropy reduction with the increased fatigue level. Indeed, during such a fatigue exercise, it has been reported that MU firing rate synchronization increases continuously until exhaustion [43]. This change can reduce variations in the sEMG signals as reported in the study by Gabriel et al. [44].

This observed entropy declination with fatigue is also in agreement with another study presented in [10]. The experimental setup described in [10] was similar to the current study: the same muscle model, isometric contraction, different levels of strength and fatigue test, whereas the method employed to measure entropy was the multiscale Sample Entropy, which differs from the methods considered in the current paper.

However, the decrease in entropy reported in [10] was only observed from the last part of the sustained exercise. In our current study, this decrease started at the beginning of the muscular exercise, and this dynamic, therefore, seems to be more in line with the dynamic of electrophysiological disturbances noted at the beginning of sustained force activities [40].

There are two possible reasons for the difference observed between dynamics of the entropy decline in [10] and in our current paper. First, the numerical stability of the Sample Entropy is sensitive to the tolerance parameter r=0.6 and the embedding dimension d=2 . These parameters were exhaustively obtained through a grid search optimization of a probabilistic cost at scale 1 ( m=1 ). Higher scales were not explored.

The second noticeable reason comes from the additional data preprocessing in [10]: the raw data acquired at a frequency sampling 1000 Hz were downsampled by a factor four to yield an effective sampling rate of 250 Hz, and the downsampled data were filtered using a lowpass filter. This implies that the authors did not explore the totality of the spectral bandwidth 0–500 Hz of sEMG signals as we did.

Now, regarding the considered Multiscale PE (left columns of Figure 3 and Figure 4), we found that two regimes stand out: an increasing entropy for low time scales and an approximately constant entropy (a plateau) for higher time scales. Other studies have also reported the presence of such regimes when using multiscale entropy [10,16,23].

In our study, the critical time scale in between the two regimes can be roughly identified in the range of 60 to 80 for the considered sEMG data sets. Given the sampling frequency of 10 kHz, this is equivalent to a time value in the range of 0.006 to 0.008 s. This time value is coherent with the study presented in [16] where the authors employed a multiscale Renyi entropy and highlighted the beginning of a plateau for time scales beyond 0.01 s. The study in [16], however, addressed the endurance of the low back muscles *erector spinae* using sEMG signals recorded during a modified version of the isometric fatigue test.

In [10], the critical time scale was identified as the intersection of two piecewise-linear regression of the multiscale sample entropy, and this was approximately equal to m≈13 , which corresponds to a time value equal t 0.05 s given the 250 Hz sampling frequency.

## 5. Conclusions

This paper addresses a fatigue study using MPE and its refinements (cMPE, rcMPE and rcDPE) for characterizing the complexity behavior of EMG signals.

The rcDPE, which theoretically exhibits the smallest bias and variance with uncorrelated noise signals according to [27], performed better in differentiating the fatigue level. This was confirmed in the present study where the highest variations in sEMG entropy were the values during a fatiguing exercise of the *biceps brachii* voluntary static contraction maintained at 70% of MVC until exhaustion.

Most importantly is the scale *m* = 10 value at which the rcDPE variations were the highest. This scale value is equivalent to down-sampling data from 10 to 1 kHz leading to the conclusion that limiting the observed data in their frequency band of interest (in the [0;500] Hz in the case of sEMG data) provides higher sensitivity. Moreover, the changes in the entropy values as a function of fatigue level that we observed are in agreement with the existing literature dedicated to fatigue studies [10,23].

From our findings, we can make some recommendations for the general use of multiscale entropy estimators in the frame of sEMG fatigue studies: The rcDPE method is recommended because it cancels correlation artifacts introduced by the coarse-graining procedure of multicale procedure. The choice of the τ parameter bringing the sEMG signal into its full frequency bandwidth prevails for concentrating data information, without any information loss, thus, yielding higher sensitivity to the complexity measures.

We thus propose researchers follow these guidelines that promote good practices in the use of MPE complexity measures. The final objective is to correctly and better exploit the potential of such measures in letting real data express their inner complexity. Lastly, the biological interpretation of the time scale deserves further comments.

We know the information content inside sEMG lies in the range of 20–500 Hz [39], which is the most appropriate sampling frequency for measurements is $f_{s}=1000$ Hz (due to Shannon’s sampling theorem), as shown by the Fatigue signal dataset. By having the original signal sampled at $f_{s}=10$ kHz with scale m=10 (or τ=10 ), we captured only the desired range where the information is meaningful.

This study can be extended to other entropy estimators with refined composite downsampling versions in order to cancel the artificial correlations between composite signals introduced by to the composite coarse graining procedure independently of the natural autocorrelation of the data. The behaviors of these entropy estimators to composite downsampling procedures are worthy of study in this experimental frame.

## Abbreviations

The following abbreviations are used in this manuscript:

PE	Permutation Entropy
MSE	Multiscale Entropy
MPE	Multiscale Permutation Entropy
cMPE	composite Multiscale Permutation Entropy
rcMPE	refined composite Multiscale Permutation Entropy
cDPE	composite Downsampling Permutation Entropy
rcDPE	refined composite Downsampling Permutation Entropy
sEMG	surface ElectroMyoGraphy
MU	Motor Unit
MUAP	Motor Unit Action Potential
MVC	Maximal Voluntary Contraction
ANOVA	ANalysis Of VAriance
